# Gender Differences in Patients with Gastric Adenocarcinoma

**DOI:** 10.3390/jcm13092524

**Published:** 2024-04-25

**Authors:** Yujin Xing, Hiroko Hosaka, Fumitaka Moki, Shota Tomaru, Yuki Itoi, Keigo Sato, Yu Hashimoto, Hirohito Tanaka, Shiko Kuribayashi, Yoji Takeuchi, Kazue Nagai, Toshio Uraoka

**Affiliations:** 1 Department of Gastroenterology and Hepatology, Gunma University Graduate School of Medicine, 3-39-15 Syowa-machi, Maebashi 371-8511, Gunma, Japan; m2120005@gunma-u.ac.jp (Y.X.); hhosaka@gunma-u.ac.jp (H.H.); s-tomaru@gunma-u.ac.jp (S.T.); yuki.itoi@gunma-u.ac.jp (Y.I.); k.sato@gunma-u.ac.jp (K.S.); y_hashi@gunma-u.ac.jp (Y.H.); h-tanaka@gunma-u.ac.jp (H.T.); shikokuri@gunma-u.ac.jp (S.K.); yoji.endoscopy@gunma-u.ac.jp (Y.T.); 2 Gunma Health Foundation, Gunma Prefectural Cancer Registry, 16-1 Horinoshita-machi, Maebashi 371-0005, Gunma, Japan; moki@gunma-hf.jp; 3 Gunma University Center for Food Science and Wellness, 4-2 Aramaki-machi, Maebashi 371-8510, Gunma, Japan; kazue-nagai@gunma-u.ac.jp

**Keywords:** gastric cancer, gender differences, adenocarcinoma, retrospective study

## Abstract

**Background**: Gastric cancer (GC) epidemiology and outcomes vary by gender. **Methods**: We reviewed 18,436 GC patients from 2008 to 2018 and looked for gender differences in clinical characteristics and survival. **Results**: The gender proportion was 71% male and 29% female. Males had a significantly (*p* < 0.001) higher proportion of differentiated GC (66.3%) and a lower proportion of undifferentiated GC (26.3%). Diagnosis through medical check-ups was more common in males (30.0% vs. 26.4%, *p* < 0.001). Clinical staging revealed 54.6% of males and 52.9% of females had localized disease without lymph node metastasis (LNM), while distant metastasis occurred in 17.4% of males and 16.9% of females (*p* < 0.001). Kaplan–Meier survival curves indicated females had a significantly higher overall survival (*p* = 0.0018). The survival advantage for females was evident in the early stages, with a significant difference in localized disease without LNM (*p* < 0.001) and localized disease with LNM (*p* = 0.0026, log-rank test) but not in the advanced stages. Multivariate Cox regression analysis showed a significantly reduced mortality risk in females (*p* < 0.001). **Conclusions**: Significant gender differences exist with regard to pathological type, presentation, clinical stage, and overall survival. These findings suggest gender-specific strategies for screening, diagnosis, and treatment.

## 1. Introduction

Gastric cancer (GC), one of the most common and deadly cancers worldwide, was responsible for over one million new cases in 2020 and an estimated 769,000 deaths, ranking fifth for incidence and fourth for mortality globally [1,2] despite a progressively falling incidence [2]. Epidemiological studies have revealed significant disparities between men and women with regard to the incidence and risk variables for GC. According to a previous study, males had a higher incidence of gastric adenocarcinoma than females, with a male-to-female ratio ranging from 2:1 to 3:1 [3]. In Japan, gastric cancer remains a significant health concern, with national incidence rates reported by the National Cancer Registry indicating that in 2019 [4], there were 124,319 new cases (85,325 males and 38,994 females). The picture is more mixed when it comes to the relationship between gender and survival. Some investigators have indicated that there are no differences in survival by sex, whereas others report better survival for women [5,6,7]. Yet, others have found females have better survival rates after treatment [8,9,10]. There are several risk factors for GC, including infection with *Helicobacter pylori*, hormonal variables, and lifestyle factors such as smoking, alcohol usage, and dietary habits; all these factors contribute to gender-specific differences [3].

This retrospective study was designed to further understand the gender-specific features of gastric adenocarcinoma, the most common type of GC, by analyzing differences in clinical features and survival between male and female patients. The results herein may be used to inform future research and clinical treatment.

## 2. Materials and Methods

### 2.1. Subjects

We reviewed the population-based cancer registry of Gunma Prefecture and corrected the data of patients with gastric adenocarcinoma diagnosed between 2008 and 2018. A total of 22,158 patients’ records were reviewed. We excluded patients with unusual adenocarcinoma, such as hepatoid adenocarcinoma, unclear diagnoses, and incomplete data. Data were extracted on age, gender, type of presentation, tumor location, pathological classification, clinical stage, survival status, and follow-up time. The survival days were calculated by determining the number of months survived from the date of diagnosis and the date of death, multiplied by 30.5 days. Additionally, for confirming the survival status, if a death notification had not been reported by the day of the investigation of survival, the cancer registration was continued, assuming survival until December 31st of that year. Ultimately, 18,436 patients (mean age of 72 years: range 15–105 years) were registered.

### 2.2. Pathological Classification

The histological results were classified based on the International Classification of Diseases for Oncology (Third Edition (ICD-O-3)) system and divided into differentiated and undifferentiated types. The pathological classification recruited was the modified Sugano–Nakamura classification [11]. Papillary and tubular adenocarcinomas classified in ICD-O-3 were categorized as differentiated types, whereas the poorly differentiated malignancies (signet ring cell carcinoma and mucinous adenocarcinoma) were categorized as undifferentiated types.

### 2.3. Clinical Staging

The clinical stage is detailed as follows: localized without lymph node metastasis (LNM), localized with LNM, adjacent organ invasion, and distant metastasis. If a patient had clinical features that matched two of the aforementioned categories, their case was classified as the more advanced stage.

### 2.4. Statistical Analysis

All statistical analyses were performed using R version 4.2.0 and SPSS Version 29.0.1.0 (171) software. We analyzed the impact of gender on the clinical characteristics of GC. Survival curves were constructed using the Kaplan–Meier and life table methods. Three variables of stratification were carefully considered: pathological classification, clinical stage, and age group. Differences in survival among these stratified cohorts, as well as between genders, were assessed using the log-rank test and generalized Wilcoxon test. A fixed time interval of 366 days was used to segment survival time for the annual survival rate. This stratification offered a comprehensive evaluation of survival and emphasized the role of gender on survival outcomes. Multivariate analysis using a Cox proportional hazards model was performed to identify independent predictors of survival in GC patients. This model considered age, gender, pathological classification, diagnosis detection, and clinical stage and focused on isolating the effects of these variables to determine their individual impact on survival outcomes. A *p*-value <0.05 was adopted as the statistical significance level.

The study was approved by the Internal Review Board of Gunma University Hospital (acceptance number: HS2021-273 and date of approval: 26 June 2023).

## 3. Results

### 3.1. Gender Differences in Age of Onset

The clinical features of the GC patients are shown in Table 1. In our dataset, there was clearly a higher prevalence of GC among males.

**Figure 1:**
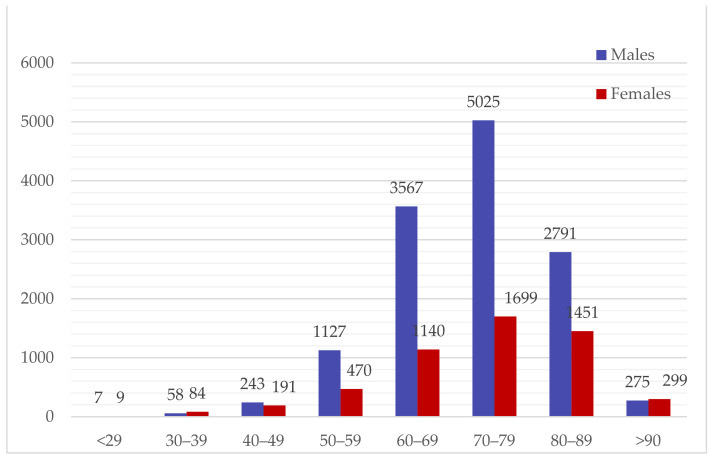
The number of the patients in each age group.

The gender proportion of the patients in each age group is shown in Figure 1. The number of males was particularly high in the middle-aged and older age groups, while the number of females was slightly higher among those aged under 40 and 90 and older. In particular, females were more likely to have GC if aged under 40; this trend was reversed with women in their 40 s.

### 3.2. Gender Differences in Pathological Type

**Figure 2:**
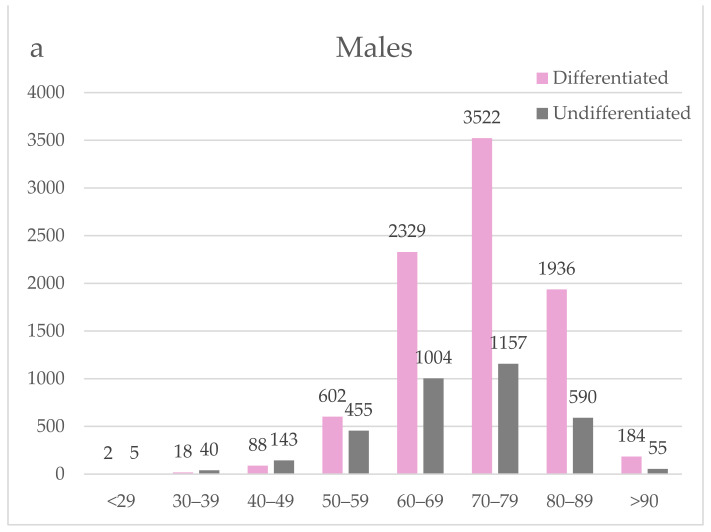

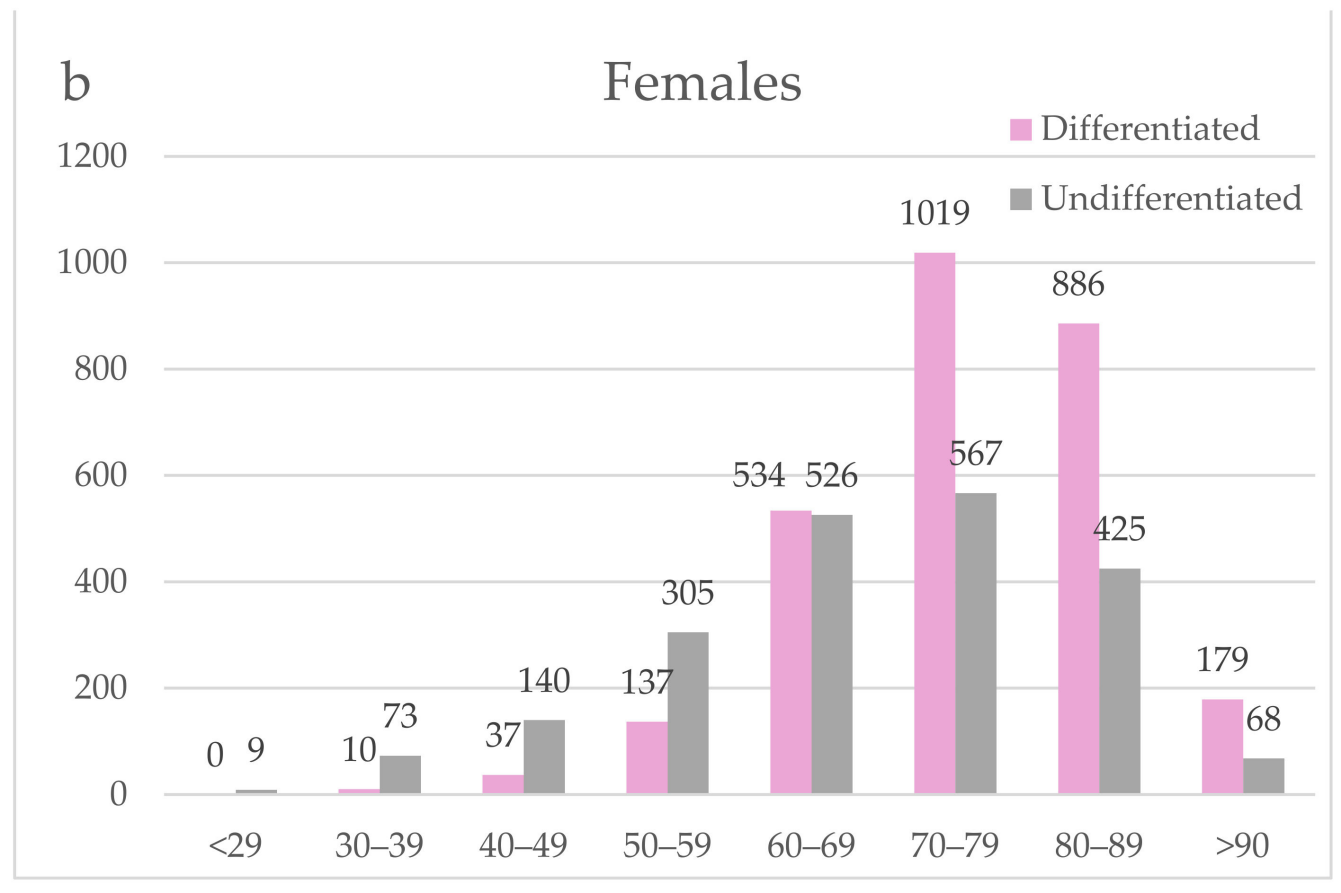
The pathological type in each age group.

Gender had a significant impact on the pathological types. Males had a higher proportion of differentiated types, with 8681 cases (66.3%) compared to females with 2802 cases (52.4%). Undifferentiated types were significantly (*p* < 0.001) less common in males, with 3449 cases (26.3%) compared to females with 2113 cases (39.5%). Figure 2 shows the proportion of the pathological type by gender and each age group. The number of differentiated types increased in males from their 50s onwards. In females, however, the number of undifferentiated types was nearly twice that of differentiated types even in women in their 50s. The incidence of differentiated types only increased only in women in their 70s or later.

### 3.3. Gender Differences in Type of Presentation and Clinical Staging

The type of presentation was also significantly different between the genders. A larger proportion of males were diagnosed during medical check-ups or cancer screenings, with 3929 cases (30.0%) vs. 1409 female cases (26.4%), which is statistically significant (*p* < 0.001). The most common stage at diagnosis was ‘localized without LNM’ in both males and females, with 7151 cases in males (54.6%) and 2827 cases in females (52.9%); this difference was statistically significant (*p* < 0.001). Localized with LNM, adjacent organ invasion, and distant metastasis stages were more prevalent in males (1389 cases, 10.6%; 1342 cases, 10.2%; and 2273 cases, 17.4%) than in females (531 cases, 9.9%; 671 cases, 12.6%; and 903 cases, 16.9%).

### 3.4. Gender Difference in Survival Rate

In this study, the median survival time was 73 months (95% CI: 69.473–76.527), with a standard error of 1.8 months. In a comparative survival analysis of GC (Table 2), gender differences were evident. Females had a higher 5-year overall survival (OS) rate at 55.3% (95% CI: 53.9–56.7) compared to males at 52.6% (95% CI: 51.7–53.5), and this advantage extended to the median OS time, with females achieving 86 months (95% CI: 76.4–95.6) versus males at 69 months (95% CI: 65.2–72.8). Pathologically, females with differentiated cancer types had a median OS of 126 months (95% CI: 114.1–137.9), which was significantly higher than males at 97 months (95% CI: 91.6–102.4). In undifferentiated cancer types, females had a better median OS of 53 months (95% CI: 40.6–65.4) than males at 27 months (95% CI: 24.6–29.3). Early clinical staging revealed a similar pattern: with LNM, the female group’s median OS was 111 months (95% CI: 78.5–143.5), surpassing that of males (65 months, 95% CI: 56.6–73.4). The prognosis worsened in both groups when there was an adjacent organ invasion or distant metastasis, and there were no gender-specific differences in OS between these groups.

**Figure 3:**
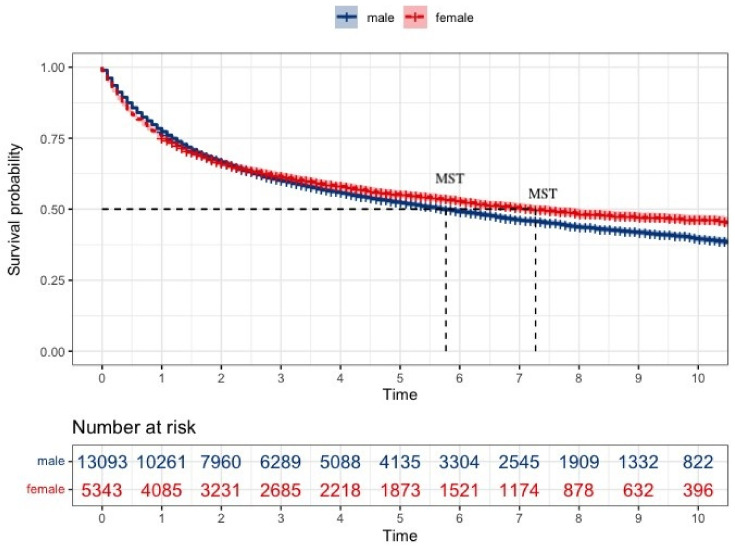
Kaplan–Meier survival curves for all patients. The dotted lines indicate MST (median survival time).

**Figure 4:**
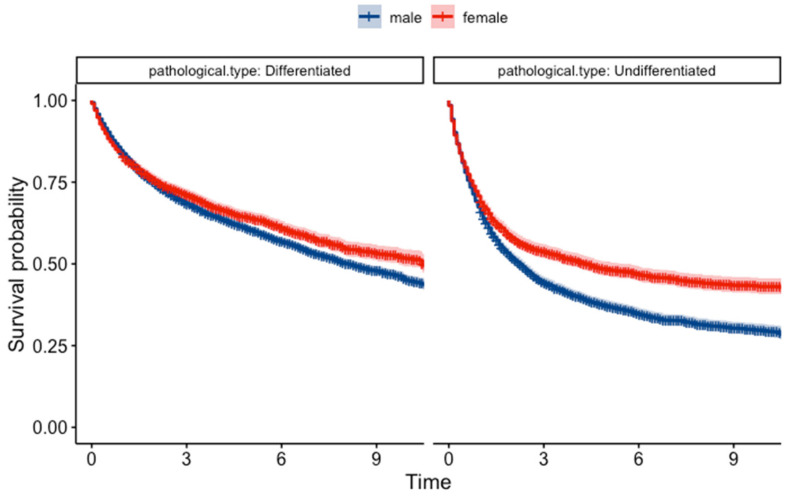
Kaplan–Meier survival curves for patients with different pathological classifications.

**Figure 5:**
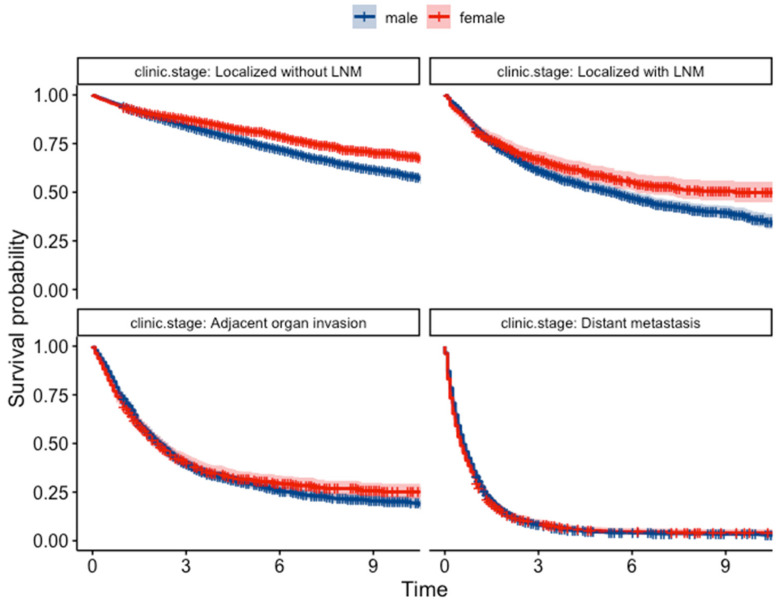
Kaplan–Meier survival curves for patients at different clinical stages.

**Figure 6:**
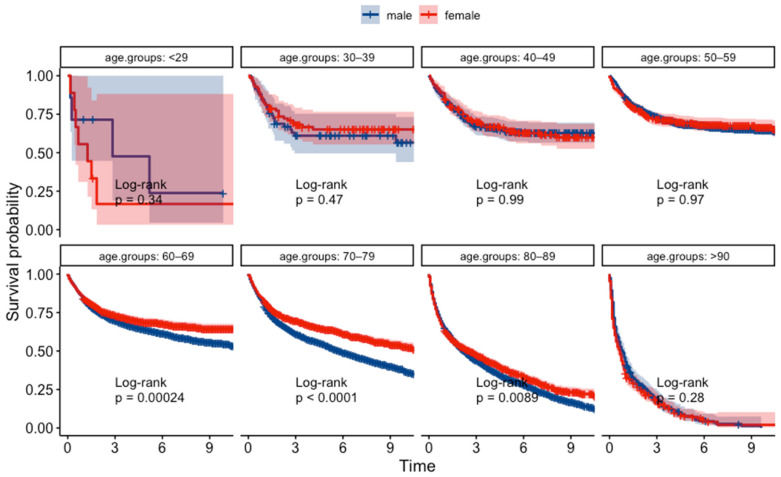
Kaplan–Meier survival curves for patients in different age groups.

The Kaplan–Meier survival analysis revealed a significant difference in survival probabilities between males and females over the study period (*p* = 0.0018) (Figure 3). Conversely, the life table analysis using the generalized Wilcoxon test did not show a significant difference (*p* = 0.756). The *p*-values from the two tests differed most likely due to differences in sensitivity between the two statistical methods. Upon further analysis of survival based on pathological classification (Figure 4), females showed a better prognosis in both differentiated types (log-rank test, *p* = 0.0025, and generalized Wilcoxon test, *p* = 0.097) and undifferentiated types (log-rank test and generalized Wilcoxon test, *p* < 0.001). The results of the gender-specific survival analysis across different clinical stages are shown in Figure 5. In patients with localized cancer without LNM (early clinical stage), females had a better survival probability than males (*p* < 0.001). A similar trend was seen in females with localized malignancies with LNM (log-rank test, *p* = 0.0026, generalized Wilcoxon test, *p* = 0.084). According to the Kaplan–Meier survival analysis, there was no statistically significant difference in survival between males and females in the younger age groups (<29, 30–39, 40–49, 50–59); Figure 6). In contrast, females had a higher survival probability in the 60–69, 70–79, 80–89 age groups, with log-rank test *p*-values of 0.00024, <0.001, and 0.0089 (generalized Wilcoxon test, *p*-values of 0.013, <0.001, and 0.488).

### 3.5. Prognostic Factors

**Figure 7:**
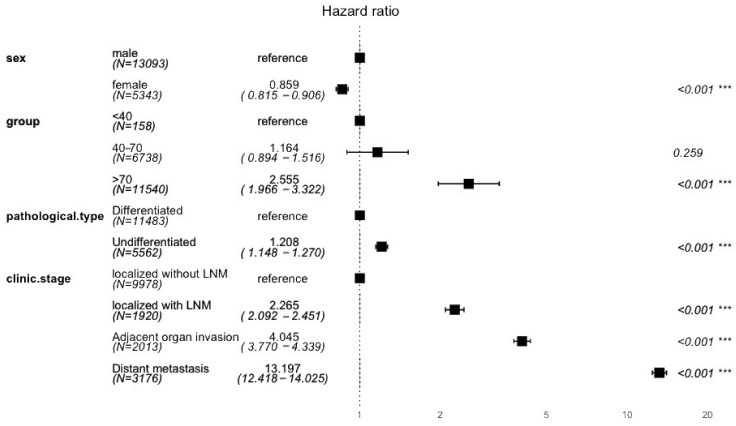
Hazard Ratios for mortality in gastric cancer by gender, age, pathology type, and clinical stage. ‘***’ indicates *p* < 0.001, which denotes a highly significant statistical difference.

We also performed a multivariant Cox regression analysis of survival data of all GC patients (Figure 7). Here, we focused on the impact of gender, age, pathological type, and clinical stage on survival time. Gender had a notable influence on survival (hazard ratio (HR) = 0.859, 95% CI: 0.815–0.906, *p* < 0.001), indicating a higher mortality risk in males. Patients aged over 70 showed a higher risk of mortality (HR = 2.555, 95% CI 1.966–3.322, *p* < 0.001) compared to the reference of patients aged under 40. The pathological type was also a significant factor (HR = 1.208, 95% CI: 1.148–1.270, *p* < 0.001), suggesting undifferentiated types are associated with an increased death risk. Clinical stage emerged as a crucial determinant, with higher stages correlating with increased mortality. Patients with localized cancer with LNM had more than double the risk of mortality (HR = 2.265, 95% CI: 2.092–2.451, *p* < 0.001), with distant metastasis presenting a more than 13-fold increased risk of mortality (HR = 13.197; 95% CI: 12.418–14.025; *p* < 0.001) compared to those without LNM. The model’s concordance index was 0.8, demonstrating good predictive accuracy, and the statistical significance was further confirmed by *p*-values less than 0.001 in the likelihood ratio test, Wald test, and score (log-rank) test.

## 4. Discussion

We observed that gender has a significant impact on GC epidemiological patterns. Consistent with a previous report [2], we observed that the incidence of GC was higher for males than for females. A deeper analysis revealed that differentiated cancers were more prevalent in males while females had a higher incidence of undifferentiated cancers. According to a previous report, there are significant gender differences in the prevalence of differentiated and undifferentiated GC types [12,13]. For example, there is a 10 to 15-year delay in the onset of intestinal-type GCs in females [14]. In addition, Furukawa et al. found that females, castrated males, and estrogen-treated male rats had a lower incidence of poorly differentiated GC [15]. This confirms that estrogens may play a protective role against GC [16,17]. This protective effect may be attributed to its ability to regulate cell growth and differentiation, inhibit inflammation, and modulate the immune response [18]. Additionally, estrogen promotes the repair of damaged DNA and suppresses the growth of cancer cells through its anti-proliferative effects [18].

The dataset used in this study did not specify the causes of death, limiting our ability to distinguish between mortality caused directly by GC and that resulting from comorbid conditions. Consequently, the survival rates reported here represent overall survival irrespective of the cause. This limitation highlights the need for future studies to include detailed cause-of-death data, which would significantly enhance the understanding of mortality risk factors in patients with GC. Like most previous studies, we found female patients had a better prognosis [10,19,20,21,22] (Figure 1). For example, the 5-year OS of female patients (Table 2) was significantly higher than that of male patients. There were additional significant differences in OS between males and females, particularly when patients were subcategorized by disease pathology, clinical stage, and age group. Moreover, a multivariate Cox regression analysis showed females had a lower risk of mortality compared to males (HR < 1).

When female patients were divided into those with early and those with advanced stage, the 5-year OS rate of the “localized without LNM” female patients was significantly higher than that of male patients. A similar trend was seen in the “localized with LNM” group. This is consistent with Bonezziti et al. who found that female patients with negative LNM at pT1 or pT2 had better survival [5]. Bando et al. also reported that gender should be taken into account as well as clinicopathological variables related to LNM when determining the appropriate therapy for early gastric cancer [23]. Our multivariate Cox regression analysis revealed that clinical stage had a significant influence on survival, reaffirming the traditional view that LNM status is one of the most important clinicopathological factors in GC prognosis. Schafmayer et al. reported that 5-year-survival in patients with advanced GC who undergo an operation with curative intent is gender-dependent; this is especially true when splenic preservation is achieved in female patients [24]. Ho et al. found that 5-year OS in advanced stage female patients with curative resection was higher than that of males [10]. While our data suggest a clear trend, the mechanisms behind these gender-specific survival advantages remain to be fully understood.

Our findings reveal a nuanced relationship between gender and survival across different age groups. There is no gender-specific impact on survival in younger age groups (<59 years), but survival was superior in older-aged (60–89 years) females than males. Although the reasons for this difference are not clear, we offer two potential explanations. First, we suggest that females tend to have better survival rates due to their higher average life expectancy. Indeed, females tend to have better prognoses when they are diagnosed with malignancies at stages that are considered curable. However, the remaining life expectancy at specific ages in Japan has been reported as 24 years in male and 29 years in female at the ages of 60 and 16 years in male and 20 years in female [25]. This remaining life expectancy was long enough that we could compare the survival rate between genders. We believe that there are still gender differences in the 5-year survival rate of those in their 60s and 70s, reflecting the favorable factors in females other than a long life expectancy.

Secondly, a decreased risk of GC is associated with longer years of fertility and postmenopausal hormone replacement therapy [26,27,28]. However, estrogen plays a tumorigenic role in the development of ERα-positive diffuse-type GC. The estrogen receptor (ER) positive rate is slightly higher in young females and in patients with poorly differentiated GC [29,30], and ER-positive patients had a bad prognosis [31]. Moreover, the gut bacterium *Helicobacter pylori* (*H. pylori*) secretes the CagA toxin, which might amplify the effects of estrogen in diffuse GC [29]. The association between ER, *H. pylori* infection, and CagA toxin may also contribute to the etiology of diffuse GC, which is more common in younger females [32,33,34,35]. As females aged 40 or younger might not have benefited from these protective factors, the survival rates of males and females in younger groups of patients are essentially equivalent.

Lastly, the study was conducted in Gunma Prefecture, which has a balanced sex ratio of 940,863 males to 959,945 females in 2023 [36], reflective of the overall Japanese population [37]. In our study, we evaluated GC outcomes within Gunma Prefecture, which is demographically representative of Japan’s national population. Furthermore, the incidence of GC in our study population coincides with the population national averages of Japan. The congruence of local demographic and disease incidence data with national statistics supports the external validity of our findings and indicates that the lessons learned from our study may be applicable to the larger Japanese context.

In conclusion, our findings enhance the understanding of gender disparities in GC outcomes. Further prospective studies are needed to elucidate the biological and environmental factors that contribute to these observed differences and to determine how these differences affect prognosis and treatment outcomes.

### Meaning of the Study

Our analysis revealed significant gender differences in clinical characteristics and survival among gastric cancer patients, underscoring the potential for refining screening strategies to be more gender-sensitive. Males exhibited a higher incidence of differentiated GC and were more frequently diagnosed through medical check-ups. This suggests the utility of developing targeted screening protocols for males.

For females, the observed survival advantage, particularly in early-stage disease, highlights an opportunity for interventions aimed at early detection. Considering this, screening recommendations for women might involve earlier initiation or the use of more sensitive screening modalities that can detect gastric cancer at its nascent stages. Furthermore, the significant survival benefit seen in women with early-stage disease underscores the critical importance of widespread educational campaigns to raise awareness about gastric cancer symptoms and the benefits of early screening.

## 5. Conclusions

Our study highlights significant gender differences in GC outcomes, indicating that gender plays a crucial role in both the epidemiology and clinical outcomes of this disease. We found that males have a higher incidence of GC and are more likely to develop differentiated types of cancer. In contrast, females generally have a higher survival rate, especially in early-stage diseases. These findings suggest the potential for gender-specific strategies in the screening, diagnosis, and treatment of GC to improve patient outcomes. Our study calls for more focused investigations to fully elucidate these differences and leverage them towards more personalized and effective approaches to GC management.

## 6. Limitations

While our study provides significant insights into gender differences in GC survival, it does have several limitations. First, our patient cohort was drawn from a single prefectural database in Japan. However, given that the incidence rates of GC observed in our cohort mirror those reported nationally, there is a potential for the extrapolation of our results to broader populations within Japan. Second, we did not have data on *Helicobacter pylori* infection status and family history, which is a critical factor in the pathogenesis of GC. However, our study is based on retrospectively available factors from a large population-based dataset. Third, our inability to differentiate the cause of death meant that we could not conclude females had a better disease-related survival. Finally, another limitation is the lack of comprehensive lifestyle data, including dietary habits, smoking, and alcohol consumption, which could interact with hormonal factors and affect cancer progression.

## Figures and Tables

**Table 1:** The clinical features of the patients with gastric cancer.

	Male	Female	*p*-Value
The number of the patients	13,093 (71)	5343 (29)	
Age (years)	71.8 (±10.1)	72.6 (±12.5)	<0.001
Tumor location			<0.001
Cardia	1359 (10.4)	374 (7)	
Fundus	586 (4.5)	190 (3.6)	
Body	5803 (44.3)	2362 (44.2)	
Antrum	4171 (31.9)	1962 (36.7)	
Unknown	1174 (9)	455 (8.5)	
Type of presentation			<0.001
Health checkup	3929 (30)	1409 (26.4)	
Accidental discovery	3114 (23.8)	1199 (22.4)	
Others	1620 (12.4)	735 (13.7)	
Unknown	4430 (33.8)	2000 (37.4)	
Pathology classification			<0.001
Differentiated	8681 (66.3)	2802 (52.4)	
Undifferentiated	3449 (26.3)	2113 (39.5)	
Unknown	963 (7.4)	428 (8)	
Clinical Stage			<0.001
Localized without LNM	7151 (54.6)	2827 (52.9)	
Localized with LNM	1389 (10.6)	531 (9.9)	
Adjacent organ invasion	1342 (10.2)	671 (12.6)	
Distant metastasis	2273 (17.4)	903 (16.9)	
Unknown	938 (7.2)	411 (7.7)	

Continuous variables were expressed as means ± standard deviation and categorical variables as numbers and percentage.

**Table 2:** The 5-Year Survival Rate and Median survival time (MST) of the patients with gastric cancer.

	5-Year OS (95% CI)		MST (Months) (95% CI)		*p*-Value	
	Male	Female	Male	Female	Log-Rank	Generalized Wilcoxon
Overall	52.6 (51.7–53.5)	55.3 (53.9–56.7)	69 (65.2–72.8)	86 (76.4–95.6)	0.0018	0.756
Pathology Classification						
Differentiated	60.7 (59.6–61.9)	64.2 (62.3–66.2)	97 (91.6–102.4)	126 (114.1–137.9)	0.0025	0.097
Undifferentiated	37.3 (35.6–39.1)	48.5 (46.3–50.7)	27 (24.6–29.3)	53 (40.6–65.4)	<0.001	<0.001
Clinical Stage						
Localized without LNM	76.3 (75.2–77.4)	81.7 (80.2–83.3)	142 (–)	–	<0.001	<0.001
Localized with LNM	51.81 (49.0–54.8)	58.88 (54.6–63.6)	65 (56.6–73.4)	111 (78.5–143.5)	0.0026	0.084
Adjacent organ invasion	29.68 (27.2–32.4)	31.6 (28.1–35.6)	26 (23.8–28.2)	25 (22.0–27.9)	0.55	0.433
Distant metastasis	4.45 (3.59–5.51)	5.23 (3.85–7.11)	7 (6.5–7.5)	6 (5.3–6.7)	0.179	0.006

OS: overall survival; MST: Median survival time.

## Data Availability

Restrictions apply to the availability of these data. Data were obtained from the Gunma Prefectural Cancer Registry and are available with permission.
